# Incidence, Risk Factors, and Epidemiology of Cystic Echinococcosis: A Complex Socioecological Emerging Infectious Disease in Khyber Pakhtunkhwa, Province of Pakistan

**DOI:** 10.1155/2018/5042430

**Published:** 2018-09-12

**Authors:** Sumbal Haleem, Sadaf Niaz, Naveeda Akhtar Qureshi, Riaz Ullah, Mansour S. Alsaid, Ali S. Alqahtani, Abdelaaty A. Shahat

**Affiliations:** ^1^Department of Zoology, Abdul Wali Khan University, Mardan, KPK, Pakistan; ^2^Department of Zoology, Kohat University of Science and Technology KUST, KPK, Pakistan; ^3^Department of Animal sciences, Qaid-i-Azam University, Islamabad, Pakistan; ^4^Department of Pharmacognosy and Medicinal Aromatic and Poisonous Plants Research Centre, College of Pharmacy, King Saud University, Riyadh, Saudi Arabia; ^5^Department of Chemistry, Government College Ara Khel FR, Kohat, KPK, Pakistan; ^6^Phytochemistry Department, National Research Center, Dokki, Giza, Egypt

## Abstract

Cystic echinococcosis is a serious zoonotic disease caused by* Echinococcus granulosus *species complex. The current study is the first attempt to determine the level of infection in domestic livestock and to explore the CE-related knowledge and awareness among livestock farmers in different districts of Khyber Pakhtunkhwa, province of Pakistan. A total of 1297 animals were examined for hydatid cysts including 538 cows, 428 buffaloes, 208 sheep, and 123 goats, at different slaughter houses in different districts of Khyber Pakhtunkhwa in 2 years from September 2015 to September 2017. For epidemiological investigations, prevalence in association with various factors (climate, age, and gender), organ specificity, types of cysts (fertile, sterile, or calcified), and viability of cysts parameters was recorded. Basing on the results obtained, areas with high prevalence were selected for further follow-ups and administration of questionnaires to the farmers and dog owners, to provide baseline data about this parasitic disease and to identify potential areas of emergence with correspondence animal and of public health significance. The finding of this study revealed the presence of CE in livestock of KP, Pakistan. The prevalence of hydatid cysts was the highest in buffaloes (15.88%) followed by cows (15.79%), sheep (15.38%), and goats (3.25%). Our investigation revealed close relationship between prevalence and animal age and gender in different months of the year. These findings also showed the highest prevalence of hydatid cysts in liver (63.49%), followed by lungs (23.80%) and mesentery (2.64%). Fertile and viable cysts were observed in all animal species except goats. The highest percentage of fertile and viable cysts was reported from the liver and lungs of sheep. For evaluation of risk factors, a total of 384 respondents were investigated. The results of current study revealed that 97.9% of farmers are not familiar with CE and transmission of this infection from dogs to human and livestock. The present study shows that CE will continue to be of medical and veterinary importance in Pakistan.

## 1. Introduction

Cystic echinococcosis (CE) is a zoonotic parasitic disease, also called “cystic hydatid disease” or hydatidosis caused by the larval stage of small tapeworms known as dog tapeworm of the genus* Echinococcus *[1]. It is characterized by the development of cysts either unilocular or may be multilocular of different extents ranging from the medium sized football to the size of a pea [2]. Genus* Echinococcus* comprises four species, i.e.,* Echinococcus multilocularis, Echinococcus granulosus, Echinococcus vogeli, *and* Echinococcus oligarthrus *[3]. There are two more* Echinococcus *species* E. ortleppi *and* E. equinus *on the basis of host-parasite interaction and their probable geographical distribution. Analysis of mitochondrial and nuclear genes of different* Echinococcus *species has led to taxonomic revisions and the genotypes G1-G3 are now grouped as* E. granulosus* sensu stricto, G4 as* Echinococcus equinus*, G5 as* Echinococcus ortleppi*, G6– G10 as* Echinococcus canadensis*, and the “lion strain” as* Echinococcus felidis* [4].* E. granulosus* life cycle is maintained by its definitive canid host, i.e., dogs that nourish the adult worm in their smaller part of their intestine while wide range of domestic livestock acts as an intermediate host. CE is responsible for extensive livestock and human mortality and morbidity [5]. This parasitic disease is listed as neglected tropical disease by World Health Organization [6].* E. granulosus* is cosmopolitan in geographical distribution and is common in South and Central regions of America, Africa, Asia, the Mediterranean region [7], United Kingdom, Australia, Europe [8], Iran, Kuwait, Iraq, Syria, Saudi Arabia, Jordan, and Pakistan's domestic animals have been found to be infected with CE [9]. Pakistan has the finest tropical dairy varieties livestock population that is well adapted to the native conditions but still the output is not as abundant as it would be [10]. Worse breeding selection, management insufficiencies, and prevalence of many parasites such as* E. granulosus *are the main reasons for this economic lose [11]. The parasitic assault is very common and is accountable for about 26.5 million (Pakistani Rupees) cost per annum to livestock sector in Pakistan, while economic losses due to* E. granulosus* in domestic animals per 100 sheep and goats were assessed as US$276.20 and for 100 infected buffaloes, cattle, and camels they were US$165.72 [12]. The epidemiology of hydatidosis varies from one area to another so control measures appropriate in one area are not necessarily of value in another [13]. It is essential to have adequate knowledge of the epidemiology of the disease before contemplating control programs [14]. To the author's knowledge, the current study is the first attempt that aimed to record the epidemiology of CE in domestic livestock in different localities of province Khyber Pakhtunkhwa, Pakistan, in addition to the determination of organ predilection for the cyst development and the fertility of cysts as well as viability of their protoscoleces. For achieving an operative CE control program, it is vital to assess the level of understanding about the awareness of disease and its preventive measure and hazardous acts that spread the infection more rapidly within the community. For these reasons, an investigation is conducted to explore the CE-related knowledge and awareness among livestock farmers in different localities of the above-mentioned province and to identify potential areas of emergence with correspondence animal and of public health significance by questionnaire based survey.

## 2. Materials and Methods

### 2.1. Epidemiological Investigation in Livestock

#### 2.1.1. Study Site

**Figure 1:**
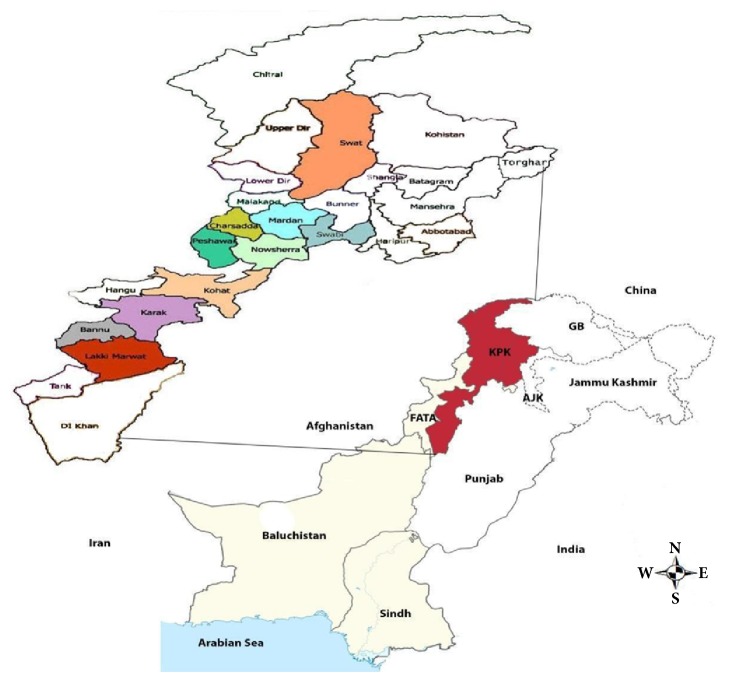
A map of Khyber Pakhtunkhwa, province of Pakistan, displaying different regions of the target area (selected districts for the study area is highlighted as well), where* E. granulosus* isolates samples were collected [15].

The province of Khyber Pakhtunkhwa (KP), previously known as North-West Frontier Province (NWFP) (Figure 1) is one of the fourth administrative province in Pakistan. It is bordered with Afghanistan to the West and is located in the northwestern area in the country. The province of KP is the third largest province of Pakistan on the basis of population and economy, although it is the smallest geographically among the four [16]. In current study about ten different districts of province KP were chosen to record prevalence of CE in domestic livestock including Peshawar, Mardan, Swabi, Nowshera, Charsadda, Swat, Kohat, Bannu, Karak, and Lakki Marwat.

#### 2.1.2. Study Design

The present study was conducted from September 2015 to September 2017, for the collection of cysts from livestock organs after slaughtering in the above-mentioned areas of KP, Pakistan.

*(a) Antemortem Examination. *Different abattoirs of various localities in KP were visited for cysts collection. These abattoirs were visited multiple times a month for collection of data regarding the prevalence of hydatid cysts in the visceral organs of slaughtered cows, buffaloes, sheep, and goat. During antemortem examination, all animals were examined for any abnormalities and the owner and place of origin were determined at the same time. At this stage, age of the particular animal was confirmed by asking the owner or where the animal was presented by the buyer (middleman), animal age was estimated by checking teeth eruption and wear (mouthing) [17], and they were conventionally grouped into three categories: < 1 year, between 1 and 5 years, and > 5 years. Animals were placed in good, medium, and poor conditions on the basis of their body conditions [18].

*(b) Abattoir Survey and the Postmortem Examination*. A total of 1297 slaughtered animals (538 buffaloes, 428 heads of cows, 208 sheep, and 123 goats) were examined. Through visual inspection and palpation of visceral organ, postmortem examination was carried out. All organs or tissues containing hydatid cysts (HC) were collected and subjected for further cyst characterization to assess their status. All organs were examined and special attention was paid to the liver, lungs, and intestine. Data related to the origin of animals, species, gender, age, cyst distribution, and observation of other diseases were recorded [19]. The data obtained from the study was subjected to statistical analysis. Basing on the results obtained, areas with high incidences were selected for further follow-ups and administration of questionnaires to the farmers and dog owners.

*(c) Examination of Cysts and Viability of Protoscoleces. *For determination of fertility, each cyst was incised or aspirate carefully and contents poured into a sterilized glass Petri dish and was observed under microscope (40X) for the hydatid protoscoleces. The germinal layer was examined for protoscoleces or broods, under microscope by keeping it in glycerine between two microscopic glass slides where it is seen as white dots. Cysts were classified as sterile, if they contain fluid without brood containing protoscoleces or calcified [20]. Viability of the protoscoleces of all fertile cysts was checked under the microscope, by observing amoeboid-like peristaltic movement (flam cell activity) [21]. Doubtful results were further examined after being stained; eosin solution (0.1% aqueous) is mixed with equal volume of hydatid cyst fluid containing protoscoleces and allowed to stand for fifteen minutes on a microscopic glass slide. The protoscoleces were classified as dead when they took up the stain and viable when they did not [22].

### 2.2. Questionnaire Survey

A structured questionnaire was developed to collect demographic information. The questions in the questionnaire were asked orally in native language of that area (usually Pashto and Urdu). In each area households were randomly selected for questionnaire administration having livestock as well as dogs, but participation was voluntary depending on the willingness of the farmers to participate in the study. Questionnaire was designed in a simple close ended way having options for ticking in order to make it easy for respondents and the latter were ask to circle their answers in an easy and understandable way. Data collected includes (i) population structure and (ii) social, ecological, and epidemiological factors which are associated with the transmission and maintenance of echinococcosis [23]. Questionnaire is provided at the end.

### 2.3. Statistical Analysis

Descriptive statistics and Chi-square test were applied in order to analyze the data with significance level of less than 0.05 using SPSS 16 software.

## 3. Results

### 3.1. Epidemiological Study

A total of 1297 animals were examined including 538 cows, 428 buffaloes, 208 sheep, and 123 goats, at different slaughter houses in successive 2 years from September 2015 to September 2017. Total infection rate was 14.57% in all animals examined (*p=*0.003). The highest prevalence was recorded in buffaloes (15.88%) and consequently followed by cows (15.79%), sheep (15.38%), and goats (3.25%). The infection rate of all slaughtered livestock in the study areas at different seasons of the years 2015-2017 is shown in Table 1.

District-wise prevalence of animals with CE indicated that the infection was the highest in Bannu district (30.76%), followed by Peshawar (18.55%), Nowshera (14.28%), Swabi (13.86%), Charsadda (12.29%), Mardan (11.90%), Lakki Marwat (10.71%), Karak (10.20%), and Kohat (9.82%) and declined to 9.24% in Swat, respectively (Table 2).

Rate of infection shows variations in the different age groups. Animals with mostly less than 1 year have less infection rate (11.29%); however, the rate of infection increases as animals aged. Table 2 shows that the highest prevalence of cysts was found in older animals of age more than 5 years (28.26%). Gender-wise distribution of the parasite indicated that higher rate of CE was observed in adult female (25.29%) livestock in comparison with male (11.97%) livestock nonsignificantly as shown in Table 2.

For the seasonal infection rate, the two years were divided into six-quarters (four months each). The data revealed (Table 3) that prevalence was the highest 59 (27.18%) in the third quarter (May, June, July, and August) in summer season, followed by 6th quarter 40 (20.30%), 5th quarter 22 (11.51%), 1st quarter 30 (10.86%), and 4th quarter 18 (9.23%), respectively. While the lowest prevalence of 20 (9.04%) was recorded in second quarter (January, February, March, and April) in spring (*p=0.003*).

**Figure 2:**
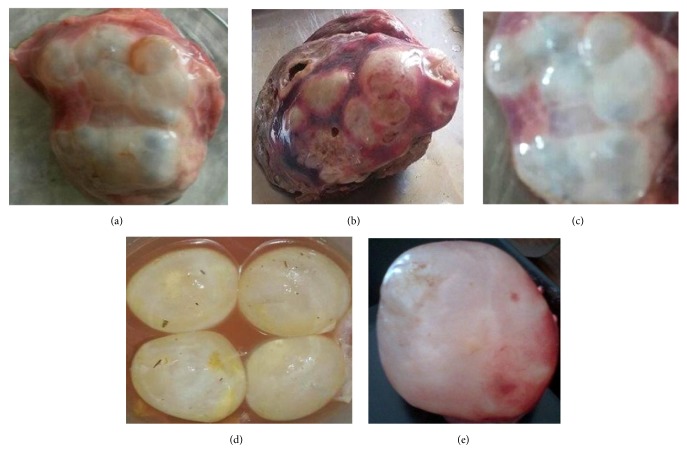
Hydatid cysts (HC) collected from various organs of slaughtered animals. (a) Liver of buffaloes. (b) Liver of cows. (c) Liver of sheep. (d) Lungs of cows. (e) Lungs of buffaloes.

Overall distribution of hydatid cysts in different organs of livestock slaughtered at different abattoirs is shown in Table 4. Out of a total of 189 animal's organs positive for hydatid cysts, 120 (63.49%) had cysts in liver, 45 (23.80%) in lungs, and 5 (2.64%) in mesentery, whereas the rest of the 19 (10.05%) infections involved heart and kidney (Figure 2). Further observations indicated that 55 (29.10%) cysts of liver and lungs had protoscoleces and hence are fertile while the rest were either sterile 101 (53.43%) or calcified 33 (17.46%). Fertility rate of the hydatid cysts collected from liver were 18 (26.08) in cows, 13 (36.11) in buffaloes, and 7 (58.33) in sheep while it was 2 (22.22), 8 (38.09), and 7 (50.00) in lungs, respectively. The findings also indicated that 6 (33.33), 4 (13.76), and 4 (57.14) cysts in liver in cows, buffaloes, and sheep had viable protoscoleces, respectively, while 2 (25.00) and 3 (50.00) cysts from lung origin had viable protoscoleces in buffaloes and sheep, respectively (Table 4).

### 3.2. Questionnaire Survey

#### 3.2.1. Sociodemographic Characteristics of the Study Population

Sociodemographic data of the study populace is very important, as to find out relevancy of the respondents to study, their approach, and practices in the related area. A total of 384 respondents were investigated in various localities of KP, Pakistan. The data in Table 5 showed that 18% respondents belonged to the age group of 18-25 years and 42.5% respondents were of the age group between 26 and 33 years. The table further describes that 27.6% respondents were of the age group between 34 and 40 years and only 10.6% respondents belonged to the age group of above 40 years, while vast majority of the study population were males (89.4%). Most of the respondents were head of the household (58.2%). The majority of the respondents mainly belong to rural area (87.6%). In addition to that, the table also indicates the family type of the respondents; 34.0% belonged to the nuclear type of family and 59.8% respondents belonged to joint type of family system. Only 5.2% respondents belonged to an extended type of family. Most of the respondents (39.4%) were having intermediate level of education. Many of the respondents (79.9%) were working as farmers as their primary occupation. Regarding the monthly income of the farmers interviewed in this study, the majority of the respondents (44.1%) have monthly income of 30,000-45,000 while only 19 (4.9%) have more than 45,000 rupees' income per month. The majority of the farmers (39.7%) investigated under this study had 11-15 years of experience in the livestock farming (Table 5).

#### 3.2.2. Household Description, General Dog, and Livestock Management

**Figure 3:**
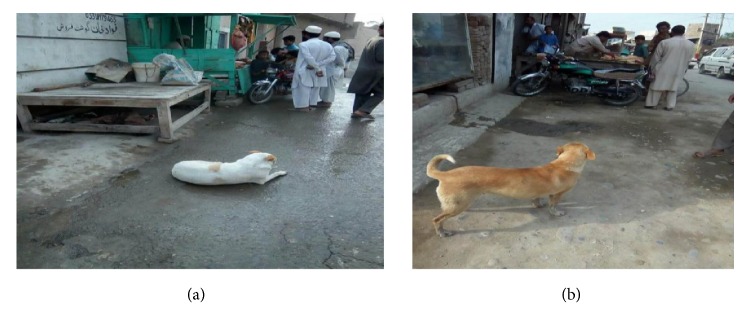
(a) and (b) show stray dogs near butcher shop in province KP (free excess to contaminated viscera of slaughtered animals).

**Figure 4:**
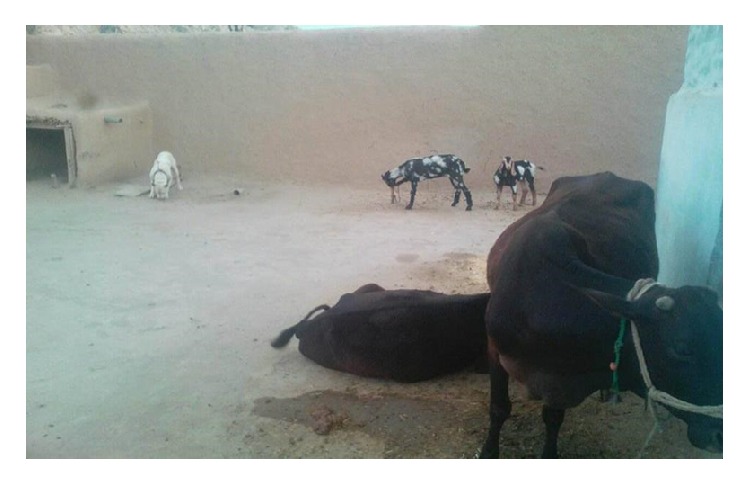
Same compound for Dog and Livestock in household is in practice in province KP.

The majority of the households (87.6%) investigated in this study resided in the rural area. Only 4.1% of respondents were migrated/refugees while all others (94.8%) were native to their districts.  Table 6 designates that most of the households (83.5%) have family members more than 5 in number. During the study, it was confirmed that 384 (100%) of the households owned at least one dog. Among the dog owners, 89.4% have one dog at their household while 9.5% have more than one dog. The survey also revealed that 82.0% of households had the living room for dogs inside their house. In addition to that 17.00% had living room for dogs inside the livestock compound. Among those that kept dogs, the majority (70.1%) of dogs were managed using the free range system and occasionally accompanied (69.3%). These dogs were mostly kept for no reason (81.1%) and security (6.2%), although some dogs were also kept for other purposes such as companionship (2.1%) and hunting (2.6%). The majority of dogs (89.7%) were aged between 7 and 11 months and were male (59.0%). In addition, 100% of respondents admitted that stray dogs were regularly spotted in their communities (Figures 3 and 4). All respondents also kept other animals at their households including 18.6% of cows, 12.6% of buffaloes, 4.1% of sheep, and 3.1% of goats and many of them kept more than one type of animals (60%). The findings also show that many of the households (50.8%) do not have proper drainage system at their area, nor proper disposal system for animal wastes (95.1%). About 71.4% of the respondents were not satisfied with the cleanliness of their environment. Most of the households (89.2%) admitted that they throw waste material outside their house. The vast majority of the respondents (80.4%) accepted that both disposable and nondisposable wastes are collected in one dustbin (Table 6).

#### 3.2.3. Practices towards CE Prevention and Possible Transmission Factors

These study findings highlighted some neglected dog management practices. Most of the households (98.5%) admitted to feeding offal to dogs and they observed dogs scavenging from abattoirs and local slaughter slabs. For example, among farmers (70.1%) who owed dogs, farmers never tied their dogs. In addition to that, 100% of the questioned farmers confirmed that they feed uncooked and raw animal flesh/viscera to their dogs. Maximum number of the farmers (69.1%) agreed that they slaughtered animals in their house in the last 12 months, as they always perform slaughtering at their house in* Eid-ul-Adha *(a religious obligation/ceremony of Muslims). Out of the total respondents (86.6%) under this investigation it was complained that it is because there was not any slaughter houses nearby. Most of the farmers (80.2%) replied that usually they leave dog fecal droppings on the land wherever they are. Majority of the farmers (68%) also confirmed that their dogs come in contact with their livestock frequently on their farms. Many of the farmers (83%) accepted that their dogs are never treated by veterinary staff when they are sick and that they never deworm their dogs. The interview findings also highlighted some unsanitary food and water management practices among the respondent farmers (Table 7).

#### 3.2.4. Knowledge and Awareness about CE Infection

*(a) Awareness of CE in Man. *40.1% of interviewees revealed that they do not have knowledge of the study subject and possibility of spreading of certain disease (zoonosis) like tapeworm diseases between animals and human. While a larger proportion (30.9%) of the surveyed households were aware of the risk of contracting rabies from dogs, fewer knew about the possibility of dogs transmitting helminth and other diseases. About 54.6% of the farmers were met in the past with people having cysts in any of their body organs despite the fact that the majority of them (96.6%) were not aware of how humans acquire that cysts. Majority of the farmers (68.6%) were self-medicated (Table 8).

*(b) Awareness of Hydatidosis in Livestock and Dogs. *This study also highlighted the fact that farmers and dogs' owners did not have knowledge and awareness of EC infection and its zoonosis from animals to human. The vast majority of the respondents (97.9%) had no idea about the proglottids in the dog's stool. None of the respondents (0.00%) had ever seen proglottids in the stool samples. On presence of cysts in organs of slaughtered livestock or those that died on their own, 34.5% of the respondents reported having seen hydatid/like cysts in one or more viscera (Table 9).

## 4. Discussion

CE is a parasitic infection of worldwide distribution, which, despite causing significant loss of health and money, is still a neglected disease [24]. A detailed knowledge of the epidemiology of this infection is important in different hosts for planning an operative control strategy [25]. To the author's knowledge, this study is the first attempt to collect and record data on CE in domestic animals and dogs in KP province of Pakistan. In the current study, the overall prevalence was the highest in buffaloes (15.88%) followed by cows (15.79%), sheep (15.38%), and goats (3.25%), respectively. These findings were similar to the findings in Maasailand, Kenya, where the highest prevalence was recorded in cattle (25.8%) followed by sheep. From different studies, although it is concluded that buffaloes have the highest prevalence, yet they have minor part in spreading in most cases sterile cysts were reported. Furthermore, cows and buffaloes are more prominent in our research as they are sold more often to slaughter than any other animal which are slaughtered at home usually. Goats have the lowest infection maybe due to the reason that goats usually ingest upper parts of the plants and shrubs unlike cows, buffaloes, and sheep that feed mostly on ground grass that might be contaminated with egg, thus increasing chance of ingestion. The prevalence was the highest in Bannu district (30.76%), followed by Peshawar (18.55%), Nowshera (14.28%), Swabi (13.86%), Charsadda (12.29%), Mardan (11.90%), Lakki Marwat (10.71%), Karak (10.20%), and Kohat (9.82%), respectively, while the lowest prevalence was recorded in Swat (9.24%). This variation in prevalence in different districts could be due to several factors including difference in geographical distribution, variation in social activities and culture, and difference in approach towards dogs as well as difference in husbandry and hygiene system [21]. In this study differences were recorded in the prevalence of CE in different age groups; those with age of more than 5 years (>5) were highly infected (28.26%) with* E. granulosus* than the age group of 1-5 years (12.36%) and less than one year (11.29%). These findings were in agreement with Azlaf and Dakkak (2006) and Regassa et al. (2010) [26, 27]. Two factors might contribute to the high prevalence in older animals. First, higher age of host reflects long duration of exposure to infection that leads to higher rate of prevalence. Second, the diagnosis of cysts in older animals is easy due to the large size of cyst [28]. In addition to that most of the slaughtered animals reported were culled because of fewer yields and were exposed to infection for long period. The prevalence of hydatid cysts was recorded higher in female animals (25.29%) than male animals (11.97%). Similar results had been observed by Banda et al. (2013) and Lemma et al. (2014) [29]. This might be due to the variation in male and female livestock management. Female livestock that produce milk are usually managed near house for the purpose of milking, which expose them more to come in contact with infected dogs. The highest prevalence was recorded in June (39.62%) while the lowest was recorded in months of December (6.89%) and October (4.25%). Monthly reports do not signify infection in that particular month due to chronic nature of hydatid disease. To acquire disease, it is difficult to put months of the year as a risk factor. These findings are in agreement with the findings of other researchers who reported a significant difference in infection rate of CE [30]. In present study, the organ livers of livestock were more observed to be infected with hydatid cysts than the organs lungs and mesentery. The results also showed that multiple organ involvement increased with increase in age including heart and kidney. These findings were in agreement with other studies, which concluded that liver is more susceptible to infection in comparison with other organs [31–34]. It might be due to the reason that liver receives the blood through bile duct with the oncosphere, after blood circulates from duodenum, and if oncosphere is not filtered in liver, it might be passed to the other organs like lungs and heart [33]. Data about the fertility and viability of hydatid cysts from livestock provide essential indicators about the transmission of CE, as they act as the main source of infection to the definitive dog host by ingestion of fertile cysts. The highest percentage of fertile cysts in the current study was found in sheep's liver (58.33%). Similarly, the highest percentage of cysts containing viable protoscoleces was reported in the liver (57.14%) of sheep. The variation in fertility, sterility, and calcification of cysts might be due to the strain differences of* E. granulosus* [35].

In this study, a total of 384 livestock farmers were given face to face questionnaire. To the best of author's knowledge, this is the first attempt in KP to record data regarding hydatid disease, its awareness, and preventive measures against such important zoonotic infection and also risk factors that contribute to the transmission of CE. Several potential risky practices had been asked in this survey to livestock farmers, especially practices associated with dog management in their household/farms. The study revealed that only 4.6% farmers tied their dogs, which reflect poor awareness among the farmers regarding the role of dogs in transmission of CE. This finding was in agreement with another study, which stated that untying dogs was a significant risk factor for the transmission of CE [36]. Moreover, most of the livestock farmer did not deworm their dogs (81.7%), and all of them fed their dogs with uncooked viscera (100%). These results are in agreement with other findings in Italy [37] and Tibet [38], which concluded that nourishing of dogs with raw offal is important risk factors for increasing public infection with this parasite. Such practices are so risky to pollute the environment with eggs of* E. granulosus* from dog feces [39]. In addition, most of the farmers exposed that their dogs (80.2%) have close association with their domestic animals and that their working dogs like hunting, guard, or sphered dogs always wander freely in the backyard, house premises, and livestock compound. The common practice (80.2%) of allowing dogs to accompany livestock while grazing on posture raises the possibility of ingestion of* E. granulosus* eggs with grass contaminated with feces of infected dogs. Unsafe water supply is also a factor involved in CE transmission due to contamination with dog feces, as some of farmers revealed that they use water from river source. The outcomes of the present study exposed that 97.9% of farmers are not familiar with hydatid disease and transmission of this infection from dogs to human and livestock. Collectively, these findings indicate that there is a crucial need to strengthen the education strategy related to health among livestock farmers and communities especially of rural areas in KPK province of Pakistan. It is quite strange that most of the farmers were against deworming their dogs, and they had not even realized that deworming the dogs would be beneficial for both human and their livestock. Furthermore, 54.6% respondents know someone in their family who had suffered or was diagnosed with cysts. Majority of the farmers has low literacy level with only 2% having some tertiary education, which indicates the ignorance regarding poor sanitary conditions that might lead to the increase in risk factors in transmission of helminthic parasites.

## 5. Conclusion and Recommendations

First and foremost, the present study results showed the prevalence of CE in domestic animals in KP province of Pakistan. This is a sign that this infection will continue to be of medical and veterinary importance in both human and livestock. The study also brought some key factors involved in the transmission which can be summarized as follows: a close association between dogs and livestock, especially in grazing area, lack of health education, easy access of dog to offal that may be infected with* E. granulosus*, no inspection of the house slaughtering, feeding of dogs with raw offal, drinking of contaminated water, presence of large number of dogs, dogs being never dewormed, and presence of fertile and viable cysts.

Therefore, with regard to high prevalence of CE, one health solution is strongly recommended for the risks posed by this parasite. Effective precautions should be adopted for controlling the risks and factors related to the transmission and spread of the disease. Applying strict measures on the offal disposal in abattoir will certainly reduce the transmission especially in developing countries like Pakistan.

## Figures and Tables

**Table 1:** Overall prevalence of hydatid cysts in slaughtered animals.

**Hosts**	**Number of Animals**		**Prevalence (**%**)**	**Chi-square ** **(X** ^**2**^ **)**	**P**
	**Examined**	**Positive**			
Cows	538	85	15.79	14.01	0.003
Buffaloes	428	68	15.88		
Sheep	208	32	15.38		
Goats	123	04	3.25		
Total	1297	189	14.57		

**Table 2:** District, age, and gender-wise prevalence of hydatid cysts in the different animal species.

**Parameters**	**Number of Animals**							**X** ^2^	**P**
	**Examined**					**Positive**	**Prevalence (**%**)**		
	**Cows**	**Buffaloes**	**Sheep**	**Goats**	**Total**				
**Districts**									

Peshawar	84	65	42	39	221	41	18.55	36.25	0.00
Mardan	60	50	30	28	168	20	11.90		
Swabi	39	40	31	27	137	19	13.86		
Charsadda	40	32	25	25	122	15	12.29		
Nowshera	39	41	27	12	119	17	14.28		
Swat	48	44	26	01	119	11	9.24		
Kohat	67	45	00	0	112	11	9.82		
Bannu	53	42	22	0	117	36	30.76		
Karak	60	38	00	0	98	10	10.20		
Lakki Marwat	48	31	05	0	84	09	10.71		

**Age**									

<1	28	23	07	4	62	07	11.29	32.33	0.00
1-5	439	382	176	54	1051	130	12.36		
>5	71	23	25	65	184	52	28.26		

**Gender**									

Male	431	362	159	92	1044	125	11.97	29.03	0.00
Female	107	66	49	31	253	64	25.29		

**Table 3:** Month wise prevalence of hydatid cysts in slaughtered animal species.

**Month**		**Animals**										**Prevalence** **(**%**)**	**(X** ^**2**^ **)**	**P**
		**Examined**					**Positive**							
		**Cows**	**Buffaloes**	**Sheep**	**Goats**	**Total**	**Cows**	**Buffaloes**	**Sheep**	**Goats**	**Total**			
1st Quarter	Sep-15	51	27	15	07	100	11	03	02	0	16	16.00	14.018	.003
	Oct-15	22	22	10	06	60	03	03	01	0	07	11.66		
	Nov-15	20	22	11	05	58	1	2	0	0	03	5.17		
	Dec-15	29	18	07	04	58	02	01	01	0	04	6.89		
2nd Quarter	Jan-16	22	15	13	03	53	0	3	02	0	05	9.43		
	Feb-16	23	21	04	05	53	02	01	0	01	04	7.54		
	Mar-16	21	20	08	05	54	1	01	02	0	04	7.40		
	Apr-16	26	20	10	05	61	5	2	0	0	07	11.47		
3rd Quarter	May-16	24	17	11	07	59	7	03	03	0	13	22.03		
	June-16	24	17	07	05	53	11	06	03	01	21	39.62		
	July-16	18	19	08	07	52	6	06	3	1	16	30.76		
	Aug-16	22	21	06	04	53	2	5	2	0	09	16.98		
4th Quarter	Sep-16	20	15	13	06	54	2	3	01	0	06	11.11		
	Oct-16	23	14	05	05	47	01	01	0	0	02	4.25		
	Nov-16	24	10	08	05	47	3	3	0	0	06	12.76		
	Dec-16	20	16	07	04	47	02	01	01	0	04	8.51		
5th Quarter	Jan-17	16	16	08	07	47	3	2	01	0	06	12.76		
	Feb-17	15	16	08	04	43	01	01	01	0	03	6.97		
	Mar-17	17	21	08	05	51	2	03	0	1	06	11.76		
	Apr-17	22	12	11	05	50	4	2	01	0	07	14.00		
6th Quarter	May-17	19	19	05	04	47	03	02	01	0	06	12.76		
	Jun-17	19	14	04	04	41	07	5	3	0	15	36.58		
	July-17	19	14	11	05	49	4	4	02	02	12	24.48		
	Aug-17	22	22	10	06	60	4	3	0	0	07	11.66		
**Total**		538	428	208	123	1297	85	68	32	04	189	14.57		

**Table 4:** Organ-wise distribution and characterization of fertile and viable hydatid cysts collected from various organs of slaughtered animal species.

**Animals**	**Infected organ Examined**	**Number of cyst examined**	**Fertile cysts examined (**%**)**	**Fertile cysts with viable protoscoleces (**%**)**
**Cows**	Liver	69	18(26.08)	6(33.33)
	Lung	9	2(22.22)	0(0.00)
	Mesentery	1	0(0.00)	0(0.00)
	Heart & Kidney	6	0(0.00)	0(0.00)

**Buffaloes**	Liver	36	13(36.11)	4(13.76)
	Lung	21	8(38.09)	2(25.00)
	Mesentery	3	0(0.00)	0(0.00)
	Heart & Kidney	8	0(0.00)	0(0.00)

**Sheep**	Liver	12	7(58.33)	4(57.14)
	Lung	14	7(50.00)	3(50.00)
	Mesentery	1	0(0.00)	0(0.00)
	Heart & Kidney	5	0(0.00)	0(0.00)

**Goats**	Liver	3.00	0(0.00)	0(0.00)
	Lung	1.00	0(0.00)	0(0.00)

**Table 5:** Sociodemographic characteristics livestock farmers (N=384) participating in CE knowledge, awareness, and practices survey in Pakistan.

**Variables**	**Category**	**Number**	%
Districts	(1) Peshawar	39	10.1
	(2) Mardan	38	9.8
	(3) Swabi	40	10.3
	(4) Nowshera	39	10.1
	(5) Charsadda	38	9.8
	(6) Swat	38	9.8
	(7) Kohat	38	9.8
	(8) Bannu	33	8.5
	(9) Karak	43	11.1
	(10) Lakki Marwat	38	9.8

Age	(1) 18-25 years	70	18.0
	(2) 26-33 years	165	42.5
	(3) 34-40 Years	107	27.6
	(4) Above 40 Years	41	10.6

Sex	(1) Male	347	89.4
	(2) Female	37	9.5

Position in the household	(1) Head of the Family	226	58.2
	(2) Dependent member in the Family	158	40.7

Area of residence	(1) Rural	340	87.6
	(2) Urban	44	11.3

Family Type	(1) Nuclear	132	34.0
	(2) Joint	232	59.8
	(3) Extended	20	5.2

Highest education level in the household	(1) Matriculation	115	29.6
	(2) Intermediate level (12 years of education)	159	41.4
	(3) Bachelor of Arts/Science (14 years of education)	40	10.3
	(4) Master of Sciences/Arts (16 years of education)	32	8.2
	(5) Any Other/none (Religious Education/Technical Education)	38	9.8

Occupation	(1) Farmers	310	79.9
	(2) Government/Private servant	74	19.1

Monthly average income (in Rupees)	(1) Below 15,000	71	18.3
	(2) 15,000-30,000	123	31.7
	(3) 30,000-45,000	171	44.1
	(4) Above 45000	19	4.9

Experience with livestock farming (in years)	(1) 1-5 years	22	5.7
	(2) 6-10 years	104	26.8
	(3) 11-15 years	154	39.7
	(4) 16-20 years	86	22.2
	(5) Above 20 years	18	4.6

**Table 6:** Descriptive results of livestock farmer's practices relevant to CE prevention.

**Variables**	**Category**	**N**	%
Household area	(1) Urban	44	11.3
	(2) Rural	340	87.6

Are you a migrant or a refugee	(1) Yes	16	4.1
	(2) No	368	94.8

Number of members of the household	(1) Below 5	26	6.7
	(2) 5	33	8.5
	(3) Above 5	324	83.5

Do you Keep Dog in House?	(1) Yes	384	100.0
	(2) No	00.0	00.0

No. of Dog(s).	(1) 1	347	89.4
	(2) More than 1	37	9.5

Living room of the Dog(s)	(1) Inside House	318	82.0
	(2) Within the livestock Compound	66	17.0
	(3) Any other/Outside home	0.00	0.00

How do you keep the dog(s)?	(1) Free range	272	70.1
	(2) Housed	94	24.2
	(3) Tied	18	4.6

How do the dogs leave the house premises?	(1) Accompanied	112	28.9
	(2) Occasionally accompanied	269	69.3
	(3) never accompanied	3.00	8.00

Reason for keeping dogs	(1) Hunting	10	2.6
	(2) Watch dog	24	6.2
	(3) Companion	8.00	2.1
	(4) No specific reason	342	88.1

Approximate ages (months)?	(1) 0-6	12	3.1
	(2) 7 -11	348	89.7
	(3) >12	24	6.2

Sex of your dog (s)	(1) Male	229	59.0
	(2) Female	131	33.8
	(3) Both	24	6.2

Are there stray dogs in your community	(1) Yes	384	100.0
	(2) No	0.00	0.001

Other species of animals kept	(1) Goats	12	3.1
	(2) Sheep	16	4.1
	(3) Buffaloes	49	12.6
	(4) Cattle	72	18.6
	(5) more than one species	235	60.0

Do you have proper drainage system in your area	(1) Yes	187	48.2
	(2) No	197	50.8

Do you have proper disposal system for animal wastes	(1) Yes	15	3.9
	(2) No	369	95.1

How do you feel the cleanliness in your local environment?	(1) Good	7.00	1.8
	(2) OK	94.00	24.2
	(3) Bad	277	71.4
	(4) Very Good	06	1.5

What kind of wastes do you find in your local environment?	(1) Human feces	00	0.00
	(2) Animal feces	76	19.6
	(3) Stagnation of wastes	196	50.5
	(4) All of these	112	28.9

After cleaning the house, what do you do with waste materials?	(1) Throw on the streets	04	1.0
	(2) throw outside the house	346	89.2
	(3) Keep it in the dustbin	16	4.1
	(4) Keep it in the garden	18	4.6

How the waste materials are collected?	(1) Both disposable and non-disposable wastes are collected in one dustbin	312	80.4
	(2) Different dust bins are used to collect disposable and non- disposable wastes	27	7.0
	(3) No dust bin is available in the street	28	7.2
	(4) Waste materials are collected from the house	17	4.4

**Table 7:** Descriptive results of livestock farmer's practices relevant to CE prevention and control.

**Variables**	**Category**	**N**	%
Do your Dog(s) consume offal?	(1) Yes	382	98.5
	(2) No	02	5.00

If yes, how is the offal prepared?	(1) Raw	384	100.00
	(2) Fried	00	00.0
	(3) Roasted	00	00.0
	(4) Boiled	00	00.0
	(5) Others	00	0.00

Have you slaughtered any livestock at home in the last 12 months?	(1) Yes	268	69.1
	(2) No	116	29.9

Where did you perform your slaughter in EidulAdha?	(1) House	268	69.1
	(2) Street	92	23.7
	(3) nearby	21	5.4
	(4) Any other	03	8.00

Is there a slaughter house nearby?	(1) Yes	48	12.4
	(2) No	336	86.6

If “Yes”, is the meat inspected done by a meat inspector?	(1) Yes	45	11.6
	(2) No	339	87.4

What do you do with livestock that die on their own?	(1) Bury	383	98.7
	(2) Burn	01	3.00
	(3) Skin and eat/sell	00	0.00

What do you do with offal of animals that die on their own?	(1) Bury	383	98.7
	(2) Burn	01	3.00
	(3) skin and eat	00	00

Do your dogs have access to dead carcasses and their viscera/offal?	(1) Yes	146	37.6
	(2) No	238	61.3

Do stray dogs have access to dead carcasses and their offal?	(1) Yes	323	83.2
	(2) No	61	15.7

Do your dogs go out to pasture with the cattle when the animals are being herded?	(1) Yes	311	80.2
	(2) No	73	18.81

Does your dog hunt small mammals in the bush when they go out?	(1) Yes	48	12.4
	(2) No	336	86.6

Do your animals graze areas where dogs defecate?	(1) Yes	264	68.0
	(2) No	120	30.9

Where does your dog usually defecate?	(1) Within the house	07	1.8
	(2) Within/ Outside house premises	30	7.7
	(3) Anywhere	347	89.4

Do children play with dogs?	(1) Yes	281	72.4
	(2) No	103	26.5

Are your dog's ever treated by veterinary staff when they are sick?	(1) Yes	00	00.0
	(2) No	322	83.0
	(3) Sometimes	62	16.0

Have your dog's ever been de-wormed?	(1) Yes	67	17.3
	(2) No	317	81.7

If “Yes” when and how often?	(1) <12 months	360	92.3
	(2) >12 months	24	6.2

Source of drinking water?	(1) River	08	2.1
	(2) Borehole	310	79.9
	(3) Well	42	10.8
	(4) Others	24	6.2

Where will you keep the water and cooked food?	(1) Open environment	03	0.8
	(2) Protected environment	11	2.8
	(3) Semi protected environment	29	7.5
	(4) either (a) (b)or(c)	341	87.9

**Table 8:** Descriptive results of livestock farmers (N=314) knowledge and awareness of way of transmission with CE in human.

**Variable**	**Category**	**N**	%
Are you aware of possible diseases/conditions that are caused by dogs?	(1) Rabies	120	30.9
	(2) Wounds from dog bite	11	2.8
	(3) Scabies	00	00.0
	(4) Worms	00	00.0
	(5) Dysentery	00	00.0
	(6) Other bacterial/viral Infections	00	00.0
	(7) Many of the diseases	253	65.2

Have you ever heard of tapeworm infections in humans?	(1) Yes	230	59.3
	(2) No	154	40.1

Have you heard or met anyone who has been diagnosed with a cyst at any hospital in the village/ your household/yourself?	(1) Yes	212	54.6
	(2) No	172	44.3

How does one know that he/she has a cyst?	(1) From people	335	86.3
	(2) Personal Observation	49	12.6

Dou you know how can people acquire a cystic infection?	(1) Yes	09	2.3
	(2) No	375	96.6

What should people with cysts infection do?	(1) Go to hospital	364	93.8
	(2) Use traditional medicine	20	5.2
	(3) Do nothing	00	00.0

Have you heard of anyone saying or complaining of the following diseases in the village?	(1) Skin nodules	29	7.5
	(2) Chronic cough	28	7.2
	(3) Ascites	00	00.0
	(4) Madness	37	9.5
	(5) Many of these	290	74.7

Have taken any de-wormer in the past one year?	(1) Yes	327	84.3
	(2) No	57	14.7

When you are sick, what did you do?	(1) go to doctor	113	29.1
	(2) self-medicated	266	68.6
	(3) do nothing	05	1.3

Did you properly take the medicine recommended by the doctor?	(1) Yes	35	9.0
	(2) No	12	3.1
	(3) Sometimes	337	87.8

Do you have a qualified Doctor in your Area?	(1) Yes	317	81.7
	(2) No	67	17.3

Do you have a well-equipped laboratory in your area, for different diagnostic tests in livestock and human?	(1) Yes	18	4.6
	(2) No	185	47.7
	(3) Yes, but far away	181	46.6

**Table 9:** Descriptive findings of livestock farmers (N=314) knowledge and awareness about transmission of CE infection in livestock and dogs.

**Variables**	**Category**	**Number**	**%**
Have you observed “proglottids” (*Echinococcus*) in dog stool?	(1) Yes	4	1.0
	(2) No	380	97.9

If “YES”, do you know what these “proglottids” are?	(1) Yes	01	0.3
	(2) No	383	98.7

If “YES”, do you know how a dog acquires this infection?	(1) Yes	01	0.3
	(2) No	383	98.7

When you see the “proglottids' in the dog stool	(1) Yes	00	00.0
	(2) No	384	99.0

Have you observed cysts in the abdominal viscera of slaughtered livestock?	(1) Yes	134	34.5
	(2) No	250	64.6

Which organs did you observe these cysts?	(1) Liver	194	50.0
	(2) Lungs	114	29.4
	(3) Mesentery	24	6.2
	(4) Heart/kidney	52	13.4

If “YES”, do you know what these “cysts” are?	(1) Yes	08	2.1
	(2) No	376	96.9

If “YES”, do you know how livestock acquire this infection?	(1) Yes	01	0.3
	(2) No	383	98.7

## Data Availability

The data used to support the findings of this study are available from the corresponding author upon request.
